# The Diverse Functions of the Calcium- and Integrin-Binding Protein Family

**DOI:** 10.3390/ijms26052223

**Published:** 2025-02-28

**Authors:** Xiaoying Wang, Zhangyi Yi, Mengwen Shi, Yu Sun

**Affiliations:** 1 Department of Otorhinolaryngology, Union Hospital, Tongji Medical College, Huazhong University of Science and Technology, Wuhan 430022, China; 2 Institute of Otorhinolaryngology, Union Hospital, Tongji Medical College, Huazhong University of Science and Technology, Wuhan 430022, China; 3 Hubei Province Clinic Research Center for Deafness and Vertigo, Wuhan 430022, China

**Keywords:** CIB, inner ear, hearing, balance, mechanoelectrical transduction (MET) channel

## Abstract

The calcium- and integrin-binding protein (CIB) family, comprising four evolutionarily conserved members (CIB1, CIB2, CIB3, and CIB4), is characterized by canonical EF-hand motifs. The functions of CIBs in the inner ear have been investigated, although further research is still necessary to gain a comprehensive understanding of them. Among the CIB family members, CIB2 is essential for auditory function. CIB3 and CIB2 jointly participate in the regulation of balance. Beyond their sensory roles, CIBs exhibit multifunctionality through calcium-dependent interactions with diverse molecular partners, contributing to the pathogenesis of various conditions, including neurological disorders, cardiovascular diseases, cancer, and male infertility. In this review, we discuss the conserved structure of the CIB family, highlighting its contributions to various biological functions. We also summarize the distribution and function of the CIB family, emphasizing the pivotal roles of CIB2 and CIB3 in hearing and balance.

## 1. Introduction

The calcium- and integrin-binding protein (CIB) family consists of four members: CIB1, CIB2, CIB3, and CIB4 [1]. A common feature of this family is the presence of multiple EF-hand domains that can bind to calcium and magnesium ions [2]. In this family, calcium- and integrin-binding protein 1 (CIB1) was first identified during investigations into the function of platelet fibrinogen receptor integrin αIIbβ3 [3]. The subsequent analysis of its structure and phylogeny revealed that CIB1 belongs to a new protein family, a novel protein family, which also includes CIB2, CIB3, and CIB4. Further studies on the CIB family have revealed their broad physiological importance and diverse roles: CIB1 and CIB2 are widely expressed in humans and mice, where they play diverse roles in neural development, normal cardiac function, and other processes by binding to multiple interacting proteins. In contrast, the expression and function of CIB3 and CIB4 are more limited.

In addition to their roles in general physiological functions, certain members of the CIB family also play significant roles in sensory systems. Hearing and balance are critical sensory systems that play essential roles in animals’ survival and interaction with their environment. Studies have proved that CIB2 is essential for hearing [4,5,6,7,8]. CIB3 and CIB2 function redundantly in the vestibule to regulate balance [9,10]. Moreover, CIB2/3 may function as auxiliary subunits of the mechanoelectrical transduction (MET) channel of inner ear hair cells [11]. The absence of CIB2, or the combined absence of CIB2 and CIB3, results in abolished MET currents and the formation of abnormal stereocilia in the inner ear hair cells of mice [4,7,8,9].

In this review, we discuss the structure and evolutionary conservation of the CIB family, exploring how these characteristics may contribute to its roles. We also summarize the roles of CIBs in various physiological processes, with particular emphasis on their function in the inner ear. Understanding the structure and function of CIB family proteins is crucial for elucidating their role in human physiology and identifying potential therapeutic targets.

## 2. Structure and Evolutionary Conservation of CIB Family Proteins

### 2.1. Protein Structure and Its Influence on Function

**Figure 1:**
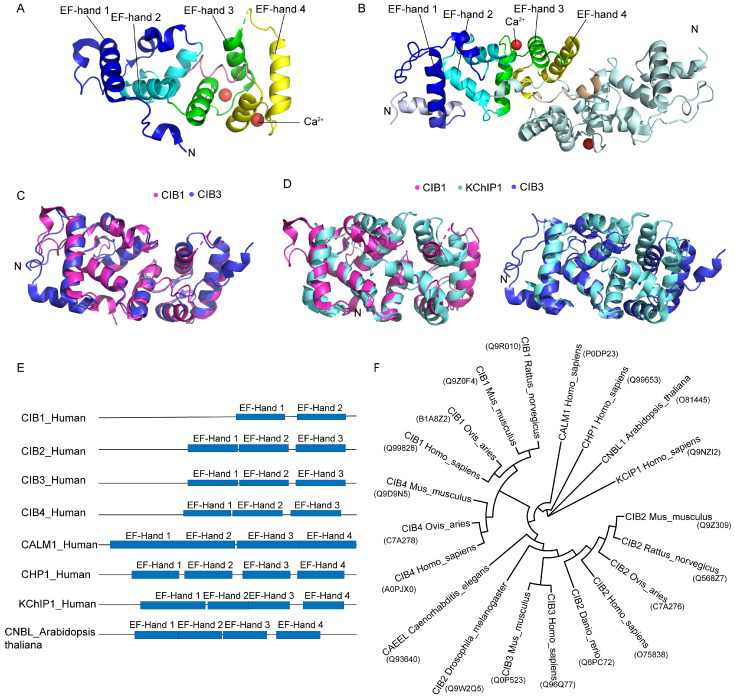
The structure and evolutionary conservation of CIBs. (**A**). The crystal structures of CIB1 (PDB code: 1XO5). The EF-hands are labeled with different colors, adapted from Gentry et al. [1]. (**B**). The crystal structures of CIB3 dimers (PDB codes: 6WU5). CIB3 forms a domain-swapped dimer, with the C-terminal residues bound in a hydrophobic trench in the partner molecule. The EF-hands are labeled with different colors, similar to Figure 1A. Adapted from Liang et al. [11]. (**C**). The superposition of CIB1 with CIB3; Root Mean Square Deviation (RMSD)= 1.118 Å. RMSDmeasures the average distance between atoms in superimposed proteins. An RMSD value under 2 Å generally indicates a high level of structural similarity, while values above 3 Å can suggest significant differences. (**D**). Structural alignment of Kv channel-interacting protein (KChIP1 or KCIP1) (PDB: 1S1E) with CIB1 (RMSD = 2.986 Å) and CIB3 (RMSD = 2.554 Å). (**E**). A structure diagram of proteins with EF-hands. EF-hands are conserved motifs. Abbreviations: Calmodulin-1 (CALM1), Calcineurin B homologous protein 1 (CHP1), Calcineurin B-like protein 5 (CNBL). (**F**). The evolutionary relationship of CIBs and other EF-hand proteins. The content in parentheses refers to the UniProt code corresponding to the protein. The dendrogram was drawn using MAGA11 and beautified using Evolview V3 [14]. Abbreviation: EF-hand domain-containing protein (CAEEL).

Currently, the crystal structures of CIB1 (protein data bank (PDB) code: 1XO5) and CIB3 (PDB codes: 6WU5, 6WU7) have been elucidated through X-ray crystallography (Figure 1A,B) [1,11]. CIB1 is monomeric during separation and interaction with targets [1], but CIB3 is a non-covalent dimer under simulated physiological conditions [12]. The sequence similarity between human CIB1 (Uniprot code: Q99828) and its homologs, CIB2 (Uniprot code: O75838), CIB3 (Uniprot code: Q96Q77), and CIB4 (Uniprot code: A0PJX0), is 59%, 62%, and 64%, respectively [1]. Certain key structural features are highly conserved (Figure 1C). For instance, these proteins share a conserved architecture composed of four EF-hand domains (EF1–EF4). EF3 and EF4, located at the C-terminal, are responsible for calcium ion binding through a classical EF-hand folding mechanism, whereas EF1 and EF2, situated in the N-terminal domain, exhibit a loss of their canonical calcium-binding capacity due to the absence of key acidic residues, such as the Asp/Glu deficiency in EF2 of CIB3 [1,13]. The EF-hand domains exhibit intricate interdomain interactions mediated by a hydrogen bond network and a hydrophobic core. For example, in CIB1, the helices H3a/H3b of EF1 and H4/H5 of EF2, along with the helices from other EF-hand domains (such as H8 and H10), form a continuous hydrophobic core that collectively stabilizes the overall conformation [11]. Additionally, all CIB family proteins have a Pro-62 residue that induces a twist between helices H3a and H3b. This structural feature is critical for maintaining the integrity of the C-terminal hydrophobic binding pocket [1].

Although CIBs share several conserved domains, they exhibit structural and functional variations due to evolutionary divergence. The crystal structure of CIB2 has not been determined; however, homology modeling suggests that Ser71 in the EF3 domain replaces the conserved Asp residue, which impairs its calcium responsiveness and suggests possible regulation by Mg^2+^ or other ions [12]. Additionally, the EF2 ring of CIB2 and CIB3, located between helices H4 and H5, contains more acidic residues compared to CIB1 [1]. These structural differences underscore the functional diversity of the CIB family in calcium signaling, while also providing a molecular basis for understanding pathogenic mutations. For example, the CIB2-R186W mutation disrupts the hydrophobic core (corresponding to Phe-173 in CIB1), which affects ligand binding or structural stability, ultimately leading to genetic deafness, such as in hearing loss [5,8].

The conserved EF-hand motifs in the C-terminal region of the CIB family play a critical role in binding Ca^2+^ and responding to fluctuations in its levels [2,15,16]. Ca^2+^ binding induces at least two kinds of structural changes: alterations in the hydrophobic binding pocket and a myristoyl switch, which involves the extrusion of the N-terminal myristoyl group from a hydrophobic cavity in the protein, thereby leading to membrane localization [1,16,17]. The hydrophobic pocket is highly conserved within the CIB family and has been implicated in ligand binding, as observed in interactions between CIB1 and integrin cytoplasmic domain αIIb [1,11]. Myristoylation is an important cellular process that typically promotes membrane binding and is crucial for the correct localization or biological function of proteins [18]. Based on structural analyses, the N-termini of CIB1, CIB2, and CIB3 are likely to undergo myristoylation, whereas the N-terminus of CIB4 does not [19]. The N-terminus of CIB1 contains a Gly2 residue, which facilitates N-myristoylation and subsequent binding to the cytomembrane [1,20]. Moreover, CIB1 acts as a Ca^2+^/myristoyl switch in cells and shuttles sphingosine kinase 1 to the cell membrane in an agonist (such as W7, a CaM-specific antagonist)-dependent manner [21]. Compared to CIB1, CIB2 undergoes N-myristoylation but lacks a Ca^2+^/myristoyl switch, and N-myristoylation does not significantly affect its intracellular localization [17]. Furthermore, CIB2 associates with the membrane through interactions with membrane lipids or proteins, rather than through N-myristoylation [17].

These structural changes enable CIBs to interact with various cellular targets and modulate downstream signaling events. For example, CIB1 can bind some key regulators, such as integrin and the inositol 1,4,5-triphosphate receptor (IP3R), thereby playing a pivotal role in regulating calcium signaling [22]. Through their regulation of calcium signaling, CIBs are involved in a range of essential cellular processes, such as cell differentiation, proliferation, and apoptosis.

### 2.2. Evolutionary Conservation

The highly conserved EF-hand motif exists in many proteins across all animal phyla and even some plant phyla, including in Kv channel-interacting protein 1 (KChIP1), calcineurin B (CnB), and calcium sensors such as calmodulin and salt hypersensitive protein 3 (SOS3) [1,13,23]. CIBs, as members of the EF-hand calcium-binding protein family, share structural and functional similarities with these proteins (Figure 1D,E). For example, the calcium-binding affinities of CIB1 are similar to those of neuronal calcium sensors (NCSs), such as KChIP1, enabling them to respond quickly at low calcium concentrations. In CIB1, the hydrophobic interface formed by EF3 and EF4 is exposed on the protein surface and resembles the ligand-binding pockets of NCS family proteins and calcineurin B, supporting their interaction with various effector proteins through hydrophobic interactions. These interactions include binding to platelet integrin αIIb and serine/threonine kinases. In addition, CIB1 shares N-terminal nutmeg modification with NCSs, which mediates its membrane localization [1].

However, unlike some NCS proteins that activate through calcium-induced dimerization or oligomerization, CIB1 maintains a monomeric state after calcium binding, regardless of ligand binding. This characteristic aligns with its unique evolutionary lineage. Phylogenetic analysis indicates that CIB1–4 constitute a distinct protein family, separate from the NCS and calmodulin families. Sequence comparison and computer modeling analysis have shown that CIB1, CIB2, CIB3, and CIB4 proteins are conserved from arthropods to humans (Figure 1C,E,F), suggesting that the CIBs play fundamental, evolutionarily preserved roles in cellular processes, such as calcium binding and integrin interaction [1,24]. Furthermore, among the four human CIBs, CIB2 and CIB3 are more similar to CIB-like proteins in *Drosophila melanogaster*, *Caenorhabditis elegans*, and *Danio rerio* [1], suggesting that CIB2 and CIB3 are more evolutionarily conserved. The evolutionary conservation of CIBs in Drosophila and other model organisms offers a robust platform for functional studies. For example, research on Drosophila has demonstrated its critical roles in phototransduction and neuronal signaling—evolutionarily conserved processes relevant to human physiology. These findings advance our understanding of CIB function and inform potential therapeutic strategies for human diseases.

CIBs are small, calcium-binding molecules that play a significant role across a broad range of organisms, including invertebrates and vertebrates. Based on sequence similarity and evolutionary conservation, these proteins may have functional redundancy. For example, in the study of platelets, it was found that the mRNA level of the CIB1 homolog CIB3 was elevated in CIB1-knockout megakaryocytes. The recombinant CIB1, CIB2, and CIB3 were specifically bound to the cytoplasmic tail peptide of αIIb [24]. In the inner ear, it has been found that CIB3 can functionally replace CIB2 in auditory hair cells in the basement membrane cultured in vitro [11]. The absence of CIB2 or CIB3 has different effects on the maintenance of stereocilia in vestibular hair cells, but it does not affect MET in vestibular hair cells [9]. However, the simultaneous loss of CIB2 and CIB3 can lead to severe defects in stereocilia and a complete loss of MET current in vestibular hair cells [9]. We have discussed this minutely in the following two parts. In fact, based on the distribution and structure of the CIB family, further research may reveal additional cooperative interactions among CIB family members.

## 3. The Diverse Functions of the CIB Family

**Figure 2:**
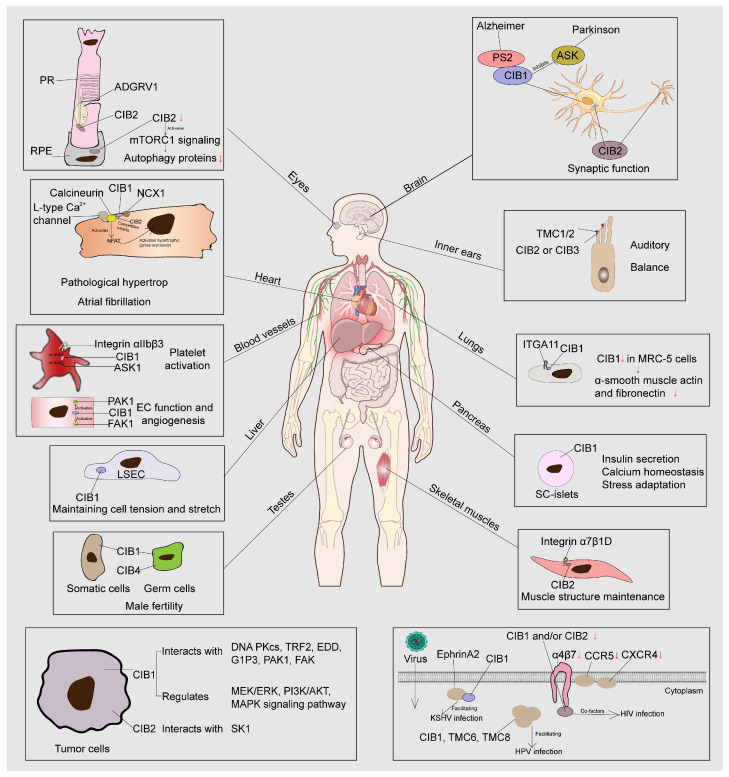
A schematic representation of the distribution and function of the CIB family. CIB family members are widely distributed and participate in various physiological processes. This diagram highlights the diverse roles and distributions of CIB family members (CIB1, CIB2, CIB3, and CIB4) across various tissues and organs, as well as their involvement in cancer and viral infections. The red arrow indicates upregulated (↑) or downregulated (↓). Abbreviations: expression.presenilin 2 (PS2), signal-regulating kinase (ASK), photoreceptor (PR), retinal pigmented epithelium (RPE), mammalian target of rapamycin complex 1 (mTORC1), Adhesion G-protein coupled receptor V1 (ADGRV1), sodium–calcium exchanger (NCX1), nuclear factor of activated T cells (NFAT), transmembrane channel-like 1 (TMC1), Integrin α11 (ITGA11), p21-activated kinase1 (PAK1), focal adhesion kinase1 (FAK1), endothelial cell (EC), liver sinusoidal endothelial cells (LSECs), stem cell-derived islets (SC-islets), DNA-dependent protein kinase catalytic subunit (DNA-PKcs), telomeric repeat-binding factor 2 (TRF2), E3 ubiquitin-protein ligase UBR5 (EDD), interferon alpha-inducible protein 6 (G1P3), phosphatidyl-inositol 3-kinase/serine-threonine kinase (PI3K/AKT), mitogen-activated protein kinase kinase (MEK)/ERK, mitogen-activated protein kinase (MAPK), sphingosine kinase 1 (SK1) C-X-C chemokine receptor type 4 (CXCR4), C-C chemokine receptor type 5 (CCR5), integrin α4β7 (α4β7), transmembrane channel-like protein 6 (TMC6), transmembrane channel-like protein 8 (TMC8),human immunodeficiency virus type (HIV), human papillomavirus (HPV), kaposi’s sarcoma-associated herpesvirus (KSHV).

CIB1and CIB2 are widely distributed in various tissues in humans and mice, while expressions of CIB3 and CIB4 are rather limited [4,25,26,27]. CIB1 is involved in several biological processes, such as hemostasis, DNA damage response, apoptosis, embryogenesis [28], mitosis [29], and spermatogenesis [30]. CIB2 is involved in various physiological processes, such as vision and hearing, and plays a role in signal transduction in platelets and skeletal muscles by interacting with integrin [19]. CIB3 is mainly expressed in the heart and liver, with relatively lower levels of expression in other tissues, such as the stomach, ovaries, testes, and muscles [25,26]. CIB3 is also expressed in the inner ear, participating in vestibular physiological function together with CIB2 [4]. CIB4 is highly expressed in the testis, playing an important role in male infertility [31]. To provide a more detailed discussion of the function of the CIB family in the inner ear, we dedicate a fourth section to explore this topic. In this section, we summarize the roles of CIB family members across other organs and tissues and their involvement in cancer and viral infections, as illustrated in Figure 2.

### 3.1. CIBs Are Associated with Neurodegenerative Diseases in the Brain

CIBs share 50% of their structural homology with neuronal calcium sensors (NCSs) such as KChIP1 (Figure 1D) [32]. CIB1 and CIB2 play pivotal roles in neuronal health, function, and survival. They are expressed in both human and mouse neurons and play a role in neurodegenerative diseases as well as NCSs.

CIB1 is mainly distributed in pyramidal neurons and interneurons of the cortex and hypothalamus (Figure 2), where it interacts with various target proteins [33]. However, in the brains of Alzheimer’s disease (AD) patients, the number of CIB1-positive neurons in these areas is significantly reduced, especially in the cortical regions [33]. CIB1 interacts with presenilin 2 (PS2) and plays a role in the pathogenesis of AD [20]. In addition, CIB1 is associated with diffuse and senile plaques in the brains of AD patients [33]. In a Parkinson’s disease mouse model, CIB1 negatively regulates dopaminergic neuron degeneration induced by 1-methyl-4-phenyl-1,2,3,6-tetrahydropyridine (MPTP) [34]. CIB1 physically interacts with apoptosis signal-regulating kinase 1 (ASK1), inhibiting the ASK1-mediated signaling cascade linked to neuronal death [34]. In addition, CIB1 interacts with pyridoxal phosphatase (PDXP), which may result in unknown nerve functions [35].

CIB2 is primarily localized in the hippocampus and cortex, especially within the Golgi apparatus of hippocampal neurons (Figure 2) [17]. This unique localization indicates the potential role of CIB2 in synaptic neurotransmission and neuronal plasticity. The N-methyl-D-aspartate receptor (NMDAR) plays a particularly important role in the potential neuronal plasticity of the central nervous system. Stimulating NMDAR and the related Ca^2+^ influx activates complex signaling pathways, leading to changes in gene transcription, translation, protein activity, and intracellular localization [36]. In cultured hippocampal neurons, neuronal activation induced an increase in CIB2 expression, especially under the activation of NMDAR [17]. The activation of NMDARs significantly upregulates CIB2 expression through Ca^2+^ influx and downstream signaling pathways involving extracellular signal-regulated kinase (ERK) 1/2 and protein kinase C (PKC) [17].

CIB1 is involved in neurodegenerative diseases and is essential for neuronal protection and signaling. In contrast, CIB2 plays a key role in synaptic function, suggesting its potential in neuroplasticity. Further research into the interaction and regulation between CIBs and other vital target proteins in the brain is essential for understanding the normal neuronal functions and the mechanisms of neurodegenerative diseases.

### 3.2. CIB2 Is Involved in mTORC1 Signaling and Autophagy in the Eyes

CIB2 is expressed in the inner and outer segments of photoreceptor (PR) cells as well as in retinal pigmented epithelium (RPE) cells (Figure 2). The functional defect of CIB2 is associated with visual defects in humans, zebrafish, and *Drosophila*. The downregulation of CIB2 in *Drosophila* significantly reduced the amplitude of light response and led to light-dependent retinal degeneration [5]. The electroretinogram (ERG) amplitude of CIB2-deficient mice showed a small but not statistically significant difference between heterozygous and homozygous mice [7], but this study only used one intensity of light for retinal function assessment and lacked controls. In the latest research, it was found that mouse CIB2 is involved in mTORC1 signaling and autophagy in the neuronal retina and retinal pigment epithelium. The deficiency of CIB2 could lead to age-related pathology, including sub-retinal pigment epithelium (RPE) deposition, the significant accumulation of drusen markers such as APOE, C3, Aβ, and esterified cholesterol, and impaired visual function, all of which can be remedied by exogenous retinoic acid [37]. In addition, in the retina, CIB2 interacts with the adhesion G protein-coupled receptor ADGRV1 (Figure 2), forming a highly overlapping protein interaction network, especially in the photoreceptor cilia, where it plays a role in maintaining the structure and function of retinal cilia [38]. CIB2 may play an important role in maintaining intracellular cilia and transport pathways [38].

### 3.3. Roles in the Heart and Blood Vessels

#### 3.3.1. The Function of CIB1 and CIB2 in Pathological Hypertrophic and Atrial Fibrillation

CIB1 is mainly localized to the sarcolemmal membrane in both mice and humans [39], where it plays a critical role in pathological cardiac remodeling (Figure 2). Under pathological hypertrophic conditions (such as chronic hypertension), CIB1 expression and membrane association are significantly upregulated [40]. CIB1 interacts with calcineurin B, the regulatory subunit of calcineurin, to anchor and coordinate calcineurin activation with L-type Ca^2+^ channel signaling. This interaction facilitates the membrane localization of calcineurin B, thereby activating the nuclear factor of activated T cells (NFAT) pathway, a key regulator of pathological hypertrophic gene expression [39,41,42]. Consistent with this fact, mice lacking CIB1 exhibited reduced calcineurin–NFAT activity and were protected from stress-induced myocardial hypertrophy, fibrosis, and dysfunction [39].

Furthermore, CIB1 contributes to atrial pathophysiology, as evidenced by its elevated expression in the right atrial myocardium of atrial fibrillation patients. Through interactions with calcineurin and the sodium–calcium exchanger (NCX1), CIB1 promotes delayed afterdepolarization and ectopic activity, thereby maintaining atrial hypertrophy and fibrillation susceptibility [43].

Building on these findings, recent studies have revealed that CIB2, as a closely related family member, also plays a critical role in cardiac pathophysiology. CIB2 expression is significantly reduced in atrial tissue from both atrial fibrillation patients and animal models. CIB2-deficient mice exhibit increased susceptibility to stress-induced AF, whereas atrial myocyte-specific CIB2 overexpression confers protection against AF development. CIB2 may attenuate atrial remodeling by inhibiting CIB1-mediated calcineurin–NFAT signaling activation, thereby suppressing this pro-arrhythmic pathway [44].

#### 3.3.2. CIB1 Regulates Platelet Function by Binding to Integrin αIIbβ3

CIB1 plays a critical role in platelet function by regulating integrin αIIbβ3 (GPIIb/IIIa)-mediated signaling (Figure 2). In vitro studies have shown that CIB1 directly interacts with the cytoplasmic domain of αIIb through its C-terminus [45], and this interaction can be enhanced in a calcium-dependent manner [27]. Upon agonist stimulation (such as with thrombin), integrin αIIbβ3 undergoes conformational activation, enabling fibrinogen binding and platelet aggregation [46]. CIB1 is particularly abundant in platelets and can bind to integrin complexes and act as a broad regulator of integrin function [47]. During platelet activation, calcium influx triggers CIB1 translocation to the cytoskeleton, where it modulates integrin-mediated cytoskeletal reorganization [27]. Studies in Chinese hamster ovary (CHO) cells revealed that focal adhesion kinase (FAK) is a downstream component of CIB-induced signaling following actin polymerization events [48].

The functional significance of CIB1 in thrombosis remains debated. While DeNofrio et al. reported no significant differences in thrombus formation, vascular occlusion, or coagulation stability in *Cib1*-knockout mice [24], Naik et al. observed prolonged tail bleeding times and delayed carotid artery occlusion in CIB1-deficient mice, suggesting impaired hemostasis [49]. These discrepancies may stem from differences in experimental conditions or analytical methods.

DeNofrio et al. also found that the mRNA expression of *Cib1* homolog *Cib3* was increased in cultured megakaryocytes isolated from *Cib1*-knockout mice [24]. In vitro, both CIB1 and CIB2 bind specifically to αIIb cytoplasmic tail peptides [24], and αIIb selectively interacts with CIB3 and CIB4 under different metal-binding conditions [2], suggesting that CIBs may have redundant or overlapping functions in the regulation of αIIbβ3.

#### 3.3.3. CIB1 Is Essential for Endothelial Cells (ECs)

Adaptive angiogenesis plays a crucial role in tissue repair and damage prevention [50], with vascular endothelial cells (ECs) serving as the central mediators of this process [51]. CIB1 emerges as a key regulator of EC function and angiogenesis, primarily through its interactions with focal adhesion kinase (FAK) [51] and p21-activated kinase (PAK) [52]. CIB1-deficient ECs display impaired angiogenic capacity, marked by reduced migration, proliferation, and tubular formation. These functional deficits correlate with the diminished activation of proangiogenic signaling mediators, including PAK1, ERK1/2, and matrix metallopeptidase 2 (MMP2), positioning CIB1 as a critical modulator of angiogenic signaling pathways [50]. Supporting this observation, *Cib1*-knockout mice exhibit significant impairments in growth factor-mediated and ischemia-induced pathological angiogenesis [51].

### 3.4. In Vitro Studies on the Role of CIB1 in the Liver

In addition to its role in vascular endothelial cells, CIB1, as a biomechanically regulated molecular factor (BMMF), plays a crucial role in the mechanobiology of liver sinusoidal endothelial cells (LSECs) in chronic liver disease (CLD) (Figure 2). CIB1 is a key factor in maintaining cell tension and stretch in response to high stiffness. The downregulation of CIB1 in LSECs in cirrhotic liver tissue may ameliorate LSEC dysfunction by regulating intracellular tone and inflammatory response, as well as by enhancing its redifferentiation [53].

### 3.5. CIB1 May Collaborate with ITGA11 in the Lungs

CIB1 and integrin α11 (ITGA11) are co-expressed in tissues such as the heart, lungs, small intestine, testes, and prostate in vivo [54,55]. The co-expression of full-length ITGA11 and CIB1 in COS7 cells, followed by immunoprecipitation experiments, confirmed this interaction [56]. Further research has found that the overexpression of CIB1 in human lung fibroblast MRC-5 cells reduces the expression of α-smooth muscle actin and fibronectin [56]. In the mouse model of pulmonary fibrosis treated with bleomycin, the expression of CIB1 in lung tissue was elevated compared to that in the control group [56]. CIB1 may synergistically regulate pulmonary fibrosis with ITGA11; however, further experimental validation is required (Figure 2).

### 3.6. The Role of CIB1 in SC-Islets

CIB1 is a key regulator of β-cell function in stem cell-derived islets (SC-islets), modulating insulin secretion, calcium homeostasis, and stress adaptation (Figure 2). Building on previous findings by Maestas et al. [57], CIB1 knockdown (KD) enhances basal and glucose-stimulated insulin secretion while elevating cytoplasmic calcium levels yet paradoxically reduces insulin content and C-peptide expression, accompanied by an elevated proinsulin/insulin ratio. Conversely, CIB1 overexpression (OE) increases insulin content without altering secretion dynamics. Under endoplasmic reticulum (ER) stress induced by brefeldin A (BFA), thapsigargin (TG), or cytokine mixtures (CMs), CIB1 KD exacerbates apoptosis, whereas OE selectively attenuates BFA-induced cell death. Transcriptomic analyses reveal that CIB1 modulates the expression of islet identity genes (such as INS, GCG, etc.) and unfolded protein response (UPR)-related genes (such as ATF4, HSPA5, etc.), with KD suppressing and OE enhancing their expression. The dual roles of CIB1 in insulin biosynthesis and ER stress resilience underscore its potential as a therapeutic target for diabetes.

### 3.7. CIB1 and CIB4 Are Related to Male Fertility

CIB1 is expressed in both somatic and germ cells during spermatogenesis in the testes (Figure 2). CIB1-knockout mice experience male infertility due to the disruption of the haploid stage of spermatogenesis. This may be related to a decrease in testicular size and the number of germ cells in the seminiferous tubules, an increase in germ cell apoptosis, and the loss of elongated sperm cells and sperm [30]. In humans, a study on sperm samples collected from infertile men in China found that the expression levels of CIB1 mRNA and protein were reduced in patients with oligoasthenozoospermia, along with increased levels of CDK1 mRNA and protein. CIB1 may be involved in the pathogenesis of oligoasthenozoospermia through the CDK1 signaling pathway [58].

CIB4 is specifically expressed in the testes of sheep, suggesting that it may play a role in male fertility [31,59]. Moreover, a high expression of CIB4 has been observed in the testes of both mice and humans. Similar to CIB1-knockout mice, *Cib4*-knockout mice exhibited impaired haploid differentiation during spermatogenesis and infertility. CIB4 was expressed during the haploid phase of spermatogenesis and was crucial for the formation of the apex region of the sperm head. In the absence of CIB4, the apex region of the sperm head failed to form a hook-like structure, which disrupted normal sperm function [26]. Together, these findings highlight the critical role of CIB1 and CIB4 in male fertility and their potential involvement in infertility.

### 3.8. CIB2 Is Highly Expressed in Skeletal Muscles

Integrin α7β1 is one of the major laminin 2 chain-binding receptors of muscle cells, mainly expressed in skeletal muscles and cardiac muscles (Figure 2), and the absence of integrin α7β1 both in humans and mice leads to myopathy [60]. CIB2 is highly expressed in the skeletal muscles of wild-type mice and localizes in the sarcolemma, tendons, and neuromuscular junctions. The gene expression of *Cib2* is reduced in the hindlimb skeletal muscles of mice lacking laminin α2 chain expression [61]. As an integrin-binding protein, CIB2 can colocalize with integrin α7β1 and may function as a cytoplasmic effector in the signaling pathway of integrin α7β1D in skeletal muscles [61]. Integrins are an important family of proteins that interact with CIB proteins, particularly CIB1 and CIB2, and may play a significant role in cell signaling pathways such as cell adhesion, migration, and survival [19,61,62,63,64].

### 3.9. Roles of CIB1 and CIB2 in Cancer

CIB1 plays an important role in promoting tumor cell cycle progression and proliferation, inhibiting tumor cell apoptosis, mediating tumor cell migration, and facilitating angiogenesis [22]. In tumor cell proliferation and survival, CIB1 could regulate oncogenic signaling pathways such as PI3K/AKT and MEK/ERK [65]. In addition, CIB1 interacts with telomerase reverse transcriptase (hTERT) to promote telomere activity and prolong telomeres, thereby maintaining the proliferation ability of cancer cells [66,67]. In terms of the process of tumor cell apoptosis, CIB1 interacts with proteins such as DNA PKcs, TRF2, and EDD, participating in DNA damage repair and helping tumor cells resist apoptosis [67,68]. CIB1 also inhibits apoptosis by regulating the MAPK signaling pathway associated with ASK1, thereby suppressing cell apoptosis [69]. Furthermore, CIB1 interacts with anti-apoptotic protein G1P3 to inhibit the depolarization of mitochondrial membrane potential and the release of cytochrome c, further reducing the occurrence of apoptosis [70]. In tumor cell migration, CIB1 enhances tumor cell migration and adhesion by interacting with various molecules such as PAK1 and FAK, thereby promoting tumor invasion and metastasis [52,71,72]. The growth of tumors is closely related to angiogenesis. In a mouse model, the absence of CIB1 significantly limits tumor growth and angiogenesis, indicating that CIB1 plays a critical role in tumor-induced angiogenesis [51,52].

CIB2 is a potential prognostic marker that is downregulated in ovarian cancer and correlates with poor prognosis. Unlike CIB1, CIB2 lacks Ca^2+^-dependent myristoyl switching but binds SK1 at the same site, inhibiting its membrane translocation and downstream signaling, including tumor necrosis factor-alpha (TNF-α)-induced apoptosis and Ras-mediated transformation [73] (Figure 2).

### 3.10. CIB1 and CIB2 in Viral Infections

- HIV

Human immunodeficiency virus type 1 (HIV-1) relies on host cell mechanisms to facilitate its replication, and it can utilize various cytokines and signaling pathways. Studies have shown that the expression levels of CIB1 and CIB2 do not affect cell survival, cell proliferation, receptor-independent virus binding to the cell surface, or the later stages of the virus replication cycle [74]. However, knockout of CIB1 and CIB2 can reduce the expression of surface molecules associated with HIV-1 infection, including CXCR4, CCR5, and integrin α4β7 (Figure 2). Moreover, the absence of CIB1 and CIB2 can also severely impair viral replication in Jurkat cells and primary CD4+ T lymphocytes, as well as weaken the envelope-mediated virus entry of X4- and R5-tropic HIV-1 [74]. CIB1 and CIB2 may be host co-factors for HIV-1 replication [74].

- HPV

Mutations in the EVER1 (epidermodysplasia verruciformis 1) and EVER2 (epidermodysplasia verruciformis 2) genes can lead to verrucous epidermal dysplasia (EV), and patients with this disease are more susceptible to human papillomavirus (HPV) infection, causing skin lesions and ultimately developing squamous cell carcinoma [75,76]. CIB1 forms complexes with EVER1 and EVER2, and the disruption of the intrinsic immunity of CIB1-EVER1-EVER2-dependent keratinocytes may contribute to the selective susceptibility to beta-HPV in patients with epidermoplasia verulosa (EV) [77]. This dysfunction in immune defense mechanisms likely underlies the increased vulnerability to HPV infections in these individuals. TMC6 and TMC8 have been identified as the genes encoding EVER1 and EVER2. Double-allele mutations in TMC6 or TMC8 were detected in over half of the cases of precancerous skin disease associated with verrucous epidermal dysplasia (EV) [76]. TMC6 and TMC8 interact with the CIB1 protein to form the TMC6-TMC8-CIB1 trimer (Figure 2) [78]. Moreover, TMC6 and TMC8 regulate CIB1 levels by protecting CIB1 from ubiquitination and proteasomal degradation. In turn, CIB1 is needed to stabilize the levels of TMC6 and TMC8, which may regulate lymphocyte function through CIB1 [78].

- KSHV

Kaposi’s sarcoma-associated herpesvirus (KSHV), also known as human herpesvirus 8 (HHV-8), infecting human endothelial cells is a key event in the development of Kaposi’s sarcoma (KS). During KSHV entry, CIB1 plays an essential role in scaffolding EphrinA2 with the cytoskeletal components myosin IIA and alpha-actin 4, facilitating viral entry into the host cells (Figure 2) [79]. This interaction underscores CIB1’s involvement in the cellular mechanisms that promote KSHV infection and contribute to the pathogenesis of KS.

## 4. Important Roles of CIB Family Proteins in the Inner Ear

**Figure 3:**
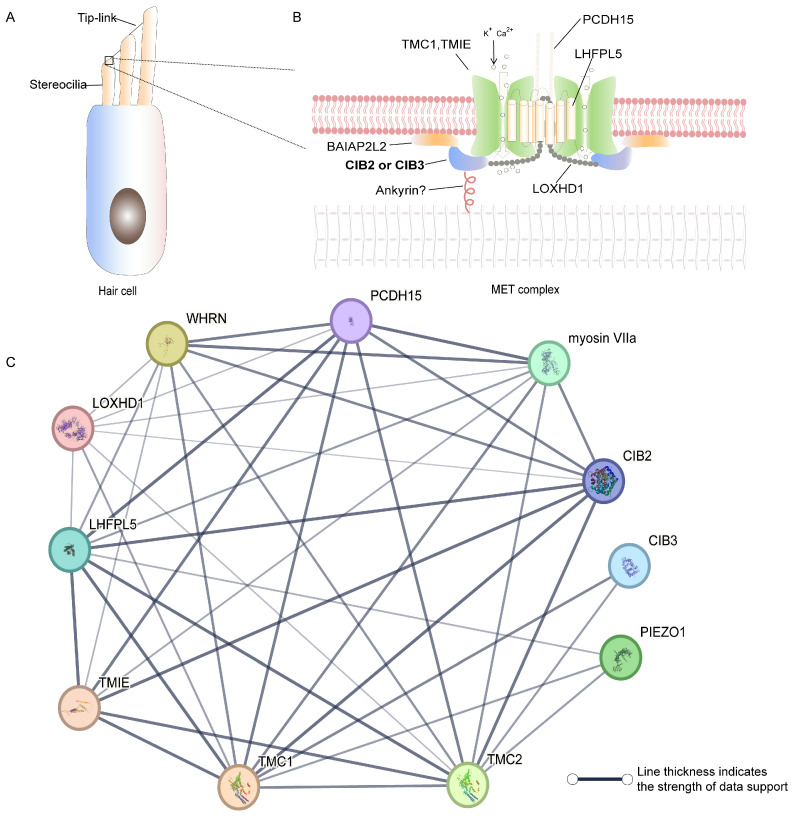
CIB2 and CIB3 as auxiliary subunits of the MET complex. (**A**): The schematic representation of inner ear hair cells and the localization of the MET complex. (**B**): The schematic diagram of the MET complex composition, illustrating the role of CIB2 and CIB3 as auxiliary subunits interacting with TMC1 and other associated proteins. The question mark after “Ankyrin” indicates that it remains to be studied whether ankyrin is the protein that connects CIB with the cytoskeleton in mice and humans. Abbreviations: BAI1-associated protein 2-like 2 (BAIAP2L2), Lipoxygenase homology domain-containing protein 1 (LOXHD1), Whirlin (WHRN), Piezo-type mechanosensitive ion channel component 1 (PIEZO1). (**C**): The interaction network of CIB2 and stereocilia-related proteins, as determined through STRING protein–protein interaction analysis.

Beyond their roles in general physiology, certain CIB family members play significant roles in sensory systems. The inner ear consists of two parts: the cochlea, which is responsible for hearing, and the vestibule, which is involved in balance. The sensory epitheliums of the inner ear have mechanosensory cells called hair cells that can detect sound or motion stimuli and transmit signals to the brain. Their apical surfaces have special cell protrusions called stereocilia. Stereocilia are actin-based and organized into rows of increasing height in mammals [80]. The mechanoelectrical transduction (MET) channel is located near the tip links of stereocilia (Figure 3A). The tip links composed of cadherin 23 (CDH23) and protocadherin 15 (PCDH15) can transmit force to the MET channel when the stereocilia are deflected, thereby transmitting sound and movement information to the central nervous system [81,82,83,84]. Some components of the MET channel have been improved, such as transmembrane channel-like 1/2 (TMC1/2), transmembrane inner ear protein (TMIE), and lipoma HMGIC fusion partner-like 5 (LHFPL5) [85,86,87,88,89,90,91,92,93]. Studies have found that CIB2 and CIB3 can interact with multiple potential MET-related proteins and serve as auxiliary subunits of the MET channel in inner ear hair cells [8,11].

**Figure 4:**
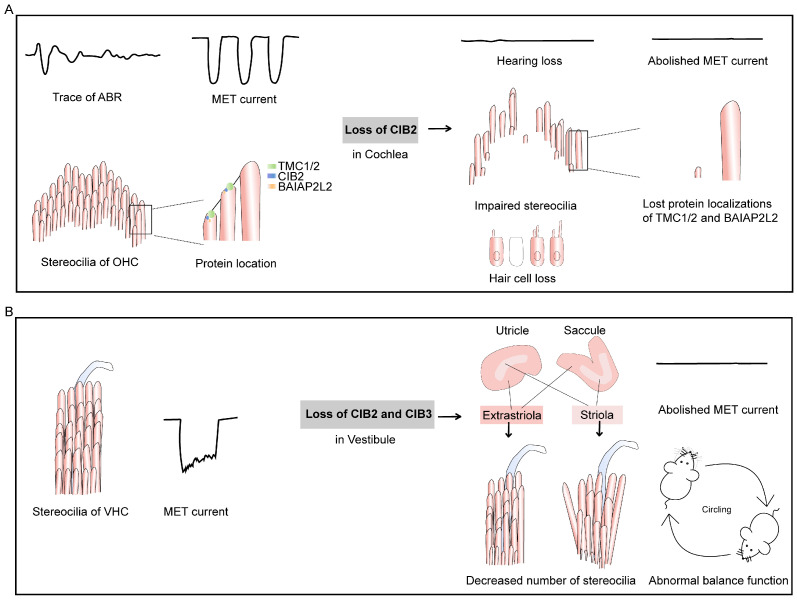
Roles of CIB2 and CIB3 in the inner ear. (**A**,**B**): Diagrams illustrating the effects of CIB2 knockout and CIB2/3 double knockout on auditory and vestibular functions in mice. (**A**): In the cochlea, the absence of CIB2 results in hearing loss, with the disruption of stereocilia and the loss of MET current. The mislocalization of TMC1 and BAIAP2L2 likely contributes to the observed cochlear abnormalities. OHC: outer hair cell. (**B**): In the vestibule, the combined loss of CIB2 and CIB3 leads to a significant reduction in the number of stereocilia, severe balance defects, and the abolition of the MET current. VHC: vestibular hair cell. These diagrams are adapted versions of diagrams from previous studies [4,7,9,91,94,95].

All *Cib* genes except for *Cib4* were detected in the inner ear [4]. A further examination revealed that *Cib1* and *Cib2* are expressed in the vestibule and cochlea, whereas *Cib3* is predominantly detected in the vestibule [4]. The absence of CIB1 or CIB3 does not impact auditory function in mice, but the loss of CIB2 does affect it [4,9]. Additionally, CIB2 and CIB3 play crucial roles in the vestibule [9,10] (Figure 4).

### 4.1. CIB2 Is Essential for Auditory Function

#### 4.1.1. Abnormal CIB2 Causes Hearing Loss

In humans, the mutations of *CIB2* are associated with hearing loss [5,6,12,19,38]. Human autosomal recessive Usher syndrome (USH) is characterized by deafness and vision loss caused by *retinitis pigmentosa*, with some patients also experiencing vestibular dysfunction [96,97,98]. Riazuddin et al. proposed that *CIB2* was associated with Usher syndrome type 1J (USH1J) and can interact with the USH proteins myosin-VIIa (MYO7A) and WHRN [5]. Subsequent research argues against this classification. It was found that CIB2 mutation only caused hearing loss, but patients showed no vestibular or retinal impairment [7,99,100]. A meta-analysis found that mutations in CIB2 were never detected in the declared cases of Usher syndrome [100]. A detailed discussion on this topic is provided in another review [19].

In contrast, murine models exhibit broader sensory deficits, including age-related vision loss [37] and mild balance issues [9] alongside hearing loss [4,7,8]. In addition, zebrafish further complicate this picture, as CIB2 loss disrupts both hair cell mechanotransduction (MET) and balance, reflecting its ancestral roles in sensory cell development [5]. These interspecies variations likely result from evolutionary divergence in sensory system redundancy and compensatory mechanisms (such as human retinal resilience to CIB2 dysfunction, with vision and other proprioceptive senses compensating for balance, making subtle balance deficits difficult to detect), as well as distinct physiological demands—such as the reliance on CIB2 for photoreceptor maintenance in mice, compared to its limited role in human vision. While CIB2’s involvement in MET and hair cell integrity is conserved, its classification as a USH protein remains uncertain.

#### 4.1.2. CIB2 Deletion Affects the Morphology of Stereocilia

The staircase architecture of the stereocilia bundle is well arranged and has a significant impact on perception. Normal stereocilia in cochlear hair cells are highly regulated by several proteins, including the scaffold protein whirlin and whirlin transporter protein myosin XVa [101]. In addition, some studies have found that MET can regulate the size of stereocilia [102,103,104,105]. In rats and mice, CIB2 is located at the tip of the stereocilia of inner ear hair cells [5,6,106]. The special location indicates that CIB2 may interact with multiple stereocilia-relevant proteins (Table 1 and Figure 3C). The loss of CIB2 results in abnormal stereocilia [4], which may be due to the abolished MET current and the changed location of the row2 protein. Some studies have proven that CIB2 can interact with the row2 proteins whirlin [5] and BAIP2L2 [107]. Recent research has found that it is the EF2 domain of CIB2 that interacts with the HDD2 region of whirlin [108]. In CIB2-knockout mice, the immunostaining of whirlin was weaker, and the localization of myosin XVa increased [108]. However, whirlin is not required for the localization of CIB2 in mouse inner ear hair cell stereocilia [5]. Moreover, immunoprecipitation and fluorescence colocalization experiments demonstrated that CIB2 can bind to the row2 component BAIP2L2. In *Cib2*-knockout mice, the stereocilia tip localization of BAIP2L2 was eliminated [107]. In mice carrying the R186W variant of CIB2, the localization of the membrane-forming protein BAIP2L2 is reduced in stereocilia [109]. The normal morphology of stereocilia and the correct localization of some row2 proteins require the presence of functional CIB2.

#### 4.1.3. CIB2 Is Part of MET Complex and Regulates MET Function

CIB2 interacts with the possible hair cell mechanical transduction complexes TMC1 and TMC2 [8]. The interaction with TMC1 may be disrupted by the N-terminal truncation of CIB2 or several other missense mutations (p.E64D, p.F91S, and p.C99W) associated with hearing loss, which located in the central region of CIB2 [8]. Another point mutation, p.R186W, disrupts one of the multiple interaction sites between CIB2 and TMC1/2, resulting in changes in electron density at the tips of stereocilia [107,109]. Moreover, the localization of TMC1/2 in the cochlear hair cells of *Cib2*-knockout mice is altered [11]. Furthermore, the latest study reported that CIB2 can interact with LOXHD1, a protein that is crucial for maintaining TMC1 as a pore subunit at the tip connection, but unnecessary for TMC2 [95]. These findings indicate that CIB2 is necessary for the localization and function of TMC1/2. In addition, CIB can form a twofold symmetric complex with two copies of TMC1 and TMIE [111]. Research on the protein structure of CIB2/3 has found that they are homologous to KChIP1 (PDB code: 1S1E) (Figure 1D), which belongs to the neuronal calcium sensor protein family and is an auxiliary subunit of the voltage-gated Kv4 channel [11]. Based on structural analysis, it was found that CIB3, as a monomer, interacts with TMC1 through its C-terminal helix, similar to the KChIP1(PDB code: 2NZ0) binding of Kv4 [11,112]. The researchers thereby infer that CIB2/3 serve as auxiliary subunits of the MET channel complex. In summary, CIB2 is necessary for the stability and channel function of MET through its interaction with TMC1, TMC2, and other related proteins (Figure 3B).

A further exploration of *C. elegans* found that evolutionarily conserved CIBs can indirectly bind ankyrin to TMC-1, connecting MET channels with the cytoskeleton and transmitting mechanical forces to TMC proteins [94]. However, it remains to be studied whether CIB is involved in the transmission of mechanical force to TMC1/2 in mouse inner ear hair cells and how this function is achieved.

In addition, in mice with mutant CIB2, the voltage-dependent reverse-polarity MET current of hair cells is still present, similar to the current observed in the *Tmc1/Tmc2* double knockout [8]. The voltage-dependent reverse-polarity MET current is regulated by tactile receptor PIEZO2, which is located in the apical surface of hair cells [113,114]. CIB2 can interact with PIEZO1 in vitro [110], but there are no reports on the interaction between CIB2 and PIEZO2. CIB2 may not participate in the PIEZO2-regulated reverse-polarity MET current.

### 4.2. CIB2 and CIB3 Work Together in the Vestibule

Among the CIB family proteins, CIB2 and CIB3 have the closest genetic relationship [1]. The injectoporation of CIB1-3 into cochlear hair cells revealed that all three proteins may be located at the tips of the stereocilia, but only the overexpression of CIB2 or CIB3 was able to partially restore MET currents in CIB2-knockout mouse hair cells [11]. According to the protein expression and mice behavior test, we propose that CIB2 and CIB3 work together to regulate balance [9]. Another research study also suggested that CIB2 and CIB3 are functionally redundant components of vertebrate transduction devices, which are necessary for MET channel activity in mouse vestibular hair cells and zebrafish mechanosensory epithelial cells [10].

#### 4.2.1. Balance Defects in Knockout Mice

*Cib2*-knockout mice showed no significant abnormalities in balance function and mechanical transduction currents in vestibular hair cells, but they were unable to stay on the rotarod for as long as their wild-type littermates, indicating that their vestibular system might have slight defects [7,115]. In our previous research, we found no difference in the rotarod performance between *Cib2*-knockout mice and wild-type mice, which may be attributed to differences in experimental methods. However, at 9 months of age, *Cib2*-knockout mice performed poorly in swimming and balance beam experiments, indicating that they do have vestibular balance dysfunction [9]. *Cib3*-knockout mice also exhibited slight abnormalities in balance function as they aged. They could not swim as well as their wild-type littermates [9]. This suggests that CIB2 and CIB3 may play a role in maintaining stereocilia over time. In *Cib2*/*Cib3*-double-knockout (DKO) mice, severe balance deficiency was observed. The DKO mice had obvious circling behavior, could not swim, and stayed on the rotarod for a long time [9]. CIB2 and CIB3 may have redundant roles in balance regulation.

#### 4.2.2. The Expression of CIB2 and CIB3 in the Vestibule

The vestibular organs include the utricle and saccule. The utricle and saccule can detect linear acceleration and have a specialized region near the center called the striola. The striola is responsible for detecting high-frequency stimuli in land-based vertebrates [116,117,118,119]. Using in situ hybridization, we showed that *Cib2* was expressed in both the striola and extrastriola of saccules and utricles, with slightly higher expression in the striola, while *Cib3* was mainly expressed in the extrastriola [9]. The expression partly explains the hypothesis that CIB2 and CIB3 may have functional overlap in the vestibule.

Vestibular type I and type II hair cells differ in their morphology and function, and there are more type I hair cells in the striola [117]. In single-cell transcriptomic analysis of the vestibular cells in humans, it was found that CIB3 was expressed in type I vestibular hair cells [120]. In the research on the Adeno-associated virus (AAV) 8-GFAP-ATOH1-induced regeneration of vestibular hair cells in mice, single-cell transcriptomic analyses revealed that CIB3 was expressed in both type I and type II vestibular hair cells, while CIB2 was mainly expressed in type II hair cells [121].

The differential expression of CIB2 and CIB3 across regions and cell types suggests subtly distinct functional roles, though their regulatory mechanisms remain unclear. Inner ear development requires the precise coordination of morphogens (such as Wnt) [122], transcription factors [123], and planar cell polarity (PCP) proteins [124]. Retinoic acid (RA), a vitamin A derivative, play a critical role in embryonic patterning, with tissue-specific levels regulated by the spatiotemporal expression of RA synthesis (ALDH1a3) and degradation (CYP26B1) enzymes [125,126]. In the inner ear, ALDH1a3 expression in the periphery and CYP26B1 expression in the striola/extrastriola establish an RA gradient essential for vestibular zone patterning. The disruption of this balance, as observed in *Cyp26b1* mutants, abolishes striolar features, highlighting the non-redundant roles of RA-metabolizing enzymes [125]. Conversely, *Aldh1a3*−/− embryos exhibit expanded striolae at the expense of extrastriolae in the maculae (utricle/saccule), while the central zones of the cristae remain unaffected [126]. *Cib2* and *Cib3* may be regulated by the RA concentration, but further research is needed to confirm this hypothesis, including investigations into whether *Cib2* and *Cib3* promoters contain RA-responsive elements (RAREs) and how RA signaling interacts with other region-specific transcription factors or epigenetic regulators.

#### 4.2.3. The Influence of CIB2/3 on Vestibular Hair Cell Stereocilia and MET Function

In mice, the individual absence of CIB2 or CIB3 had different effects on the maintenance of stereocilia: the loss of CIB2 resulted in the abnormal maintenance of stereocilia specifically in the striola, while the absence of CIB3 caused abnormal stereocilia maintenance in both the striola and extrastriola. A possible reason is that the interaction of CIB2/3 with row2 proteins is disrupted, thereby influencing the stereocilia. Abnormal stereocilia may be related to the damage of the MET function. However, the loss of CIB2 did not affect the MET current of striola vestibular hair cells (VHCs) at P6-P8. The low expression level of CIB3 in the striola may be enough for MET function. Moreover, the simultaneous loss of CIB2 and CIB3 resulted in a severe maintenance deficiency of stereocilia and the complete loss of MET current in both striola and extrastriola VHCs [9]. CIB2 and CIB3 may work together to regulate the MET function and stereocilia maintenance in the vestibule.

One research study on Tmc in the zebrafish utricle found that tmc1, tmc2a, and tmc2b have differential expression patterns in the extrastriola and striola. In turn, there are functional differences in the mechanotransduction of dynamic stimuli. Tmc2a is required for the sensitivity of utricle hair cells to a wide range of frequencies, whereas Tmc2b and Tmc1 are required for sensitivity to a lower range of frequencies [127]. Combining the distribution of CIB2 and CIB3, we assume that CIB2 and CIB3 function with TMC1 and TMC2 proteins in the mouse vestibular hair cells with a delicate arrangement. They may form different combinations in the two types of vestibular hair cells, responding to various stimuli.

## 5. Discussion

The CIB family has various functions and plays important roles in multiple processes. CIB1 and CIB2 are widely distributed. They play emerging roles in cancer and viral infections, providing a vision of a new healing directions. CIB1 regulates cancer cell migration, proliferation, and survival in vitro and promotes tumor growth in breast cancer models in vivo [65]. CIB1 has emerged as a promising therapeutic target in triple-negative breast cancer pathogenesis. Computational strategies have enabled the identification of the first small-molecule CIB1 ligand, with target engagement validated using the TR-FRET assay. This compound demonstrates selective cytotoxicity in CIB1-expressing cancer cell lines while showing negligible activity in CIB1-depleted models [128]. In MDA-MB-468 human breast cancer cells, the macrophage-mediated delivery of CIB1-targeting siRNA significantly attenuated tumor spheroid growth, accompanied by marked reductions in both CIB1 and KI67 mRNA expression levels [129]. In advanced hepatocellular carcinoma (HCC), lenvatinib, a multitarget tyrosine kinase inhibitor, is the standard treatment. AAV9-mediated CIB1 overexpression conferred lenvatinib resistance in patient-derived xenografts (PDXs), while its knockdown restored sensitivity, highlighting CIB1 as a promising therapeutic target [130]. In addition, CIB2 holds potential as a therapeutic target for overcoming epidermal growth factor receptor-tyrosine kinase inhibitor (EGFR-TKI) resistance in non-small-cell lung cancer (NSCLC). CIB2 promotes epithelial-to-mesenchymal transition (EMT) through the upregulation of zinc finger e-box binding Homeobox 1 (ZEB1), driving gefitinib resistance [131]. Moreover, the importance of CIB1 and CIB4 in male infertility may inspire research on the rise and treatment of infertility, as well as on non-hormonal male contraceptive pills.

Recently, research has increasingly focused on the roles of CIB2 and CIB3 in the inner ear, providing insights into the mechanisms of mechanoelectrical transduction involved in hearing and balance. Based on our observations, the CIB family has overlapping functions, which may result from their conservative structure. However, there remain some unanswered questions: Can the overexpression of CIB3 can prevent hearing loss in *Cib2*-knockout mice? Why do CIB2 and CIB3 express differently in the vestibule? Which genes regulate the expression of CIB2 and CIB3 in the vestibule? In future studies, we could focus on the cooperation of the CIB family, which may provide some interesting breakthroughs.

Recent advances in gene therapy have demonstrated remarkable potential for treating genetic hearing loss, with AAV-mediated gene delivery showing particular promise in preclinical models of congenital deafness [132,133]. Notably, CIB2 mutations have been genetically linked to human hereditary hearing impairment [5,99], yet the AAV-based restoration of *Cib2* or its paralog *Cib3* in *Cib2*-mutant mice remains unexplored. Significantly, the AAV-mediated delivery of *Tmc1* or *Tmc2* in the inner hair cells of *Tmc1*-knockout and Beethoven (TMC1 p.M412K) mice—models for human DFNB7/11 and DFNA36—restored auditory function [134,135,136]. Given the functional correlation between TMC proteins and CIBs in inner ear hair cells, targeted *Cib2* gene therapy warrants systematic investigation, with *Tmc1*’s therapeutic success supporting its potential for treating *CIB2*-related hearing loss.

## Figures and Tables

**Table 1:** The interaction proteins of CIB2 and CIB3 in the inner ear. FRET: fluorescence resonance energy transfer. co-IP: co-immunoprecipitation.

Interacting Proteins	CIB Family Members	Verification Methods
TMC1	CIB2, CIB3	FRET [8], co-IP [11]
TMC2	CIB2	FRET [8], co-IP [11]
PIEZO1	CIB2	co-IP [110]
BAIAP2L2	CIB2	co-IP, colocalization [107]
WHRN	CIB2	co-IP [5]
MYOSIN VIIa	CIB2	co-IP [5]
LOXHD1	CIB2	co-IP [95]
LHFPL5	CIB2	co-IP [91]

## Data Availability

No new data were created or analyzed in this study.
